# Cannabidiol at the crossroads: panacea, placebo, or problem?

**DOI:** 10.1038/s41386-026-02415-0

**Published:** 2026-04-18

**Authors:** L. Cinnamon Bidwell, Ryan Vandrey

**Affiliations:** 1 Institute of Cognitive Science, Department of Psychology and Neuroscience, University of Colorado Boulder, Boulder, CO USA; 2 Department of Psychiatry and Behavioral Sciences, Johns Hopkins University School of Medicine, Baltimore, MD USA

**Keywords:** Medical research, Drug discovery, Psychology

## Abstract

Cannabidiol (CBD) is widely perceived as a safe and effective treatment for a growing list of health indications and use for general wellness. Evidence for its safety and efficacy comes from a variety of sources, including preclinical studies, clinical trials, and observational studies of real-world evidence. The challenge in interpreting these data is that CBD products are diverse with respect to format, formulation, intended route of administration, dose, and regulatory oversight with regard to quality assurance and labeling. This *Circumspectives* article presents two perspectives: one emphasizing CBD’s potential as a pharmacologically diverse therapeutic agent with tremendous potential to treat debilitating health conditions for which there are limited alternative therapies and another highlighting concerns with the quality of existing evidence, misapplications of use, and risks related to both direct effects of CBD as well as quality control issues with retail products that lack proper regulatory oversight. Finally, these perspectives are integrated to provide guidance for reducing variability and improving clinical translation in CBD research.

## Perspective one: a low-risk therapeutic with diverse application potential in need of clinical maturity

Cannabidiol (CBD) is a phytocannabinoid derived from the *Cannabis sativa* plant, which has a diverse pharmacology that includes activity across serotonergic (5-HT1A), endocannabinoid, GABAergic, vanilloid, and other receptor systems [1]. Unlike delta-9-tetrahydrocannabinol (Δ9-THC), CBD is non-intoxicating and exhibits low abuse liability [2]. Preclinical and early-phase human trials have demonstrated anti-inflammatory, anxiolytic, neuroprotective, and antipsychotic properties, making CBD a promising candidate in multiple psychiatric and neurological domains [1].

The most robust clinical evidence to date lies in treatment-resistant pediatric epilepsy, where CBD (Epidiolex) has gained approval from multiple regulatory health agencies (e.g., U.S. Food and Drug Administration) for the treatment of rare seizure disorders such as Lennox-Gastaut and Dravet syndromes [3]. Beyond epilepsy, there is promising evidence for the application of CBD in treating psychiatric disorders. Hurd and colleagues (2019) showed that CBD reduced cue-induced craving and anxiety in individuals with opioid use disorder [4]. Similarly, Leweke and colleagues reported that CBD had antipsychotic effects comparable to amisulpride in schizophrenia, with a superior side-effect profile [5]. Emerging trials suggest potential benefits in social anxiety disorder, post-traumatic stress disorder (PTSD), substance use disorders, and other stress-related conditions [6–10].

The pharmacological basis of CBD’s potential therapeutic effects in psychiatry and behavioral health is a topic of ongoing research. CB_1_ receptors are densely expressed in brain areas associated with reward and memory, including the nucleus accumbens (NAcc), substantia nigra, and hippocampus [11]. From a pharmacological perspective, CBD’s pleiotropic mechanisms may be particularly well-suited for treating complex, comorbid conditions that do not respond adequately to existing treatments. It acts on a broad array of targets [12], including the endocannabinoid system, serotonin receptors (5-HT1A), transient receptor potential channels (TRPV1), peroxisome proliferator-activated receptors (PPARs), and adenosine signaling pathways [13, 14]. Each of these signaling systems maps onto domains of psychiatric and behavioral health that provide potential neurobiological mechanisms by which CBD might produce purported clinical benefits: 5-HT1A agonism is associated with anxiolytic and antidepressant effects; TRPV1 desensitization and modulation contribute to analgesia and attenuation of hyperalgesia; PPAR activation and adenosine signaling regulate neuroinflammatory cascades implicated in mood disorders and neurodegeneration [13, 14]. In addition, CBD can act as a negative allosteric modulator of CB1 receptors [15] and increase concentrations of the endogenous cannabinoid anandamide via inhibition of fatty acid amide hydrolase (FAAH) in a concentration-dependent manner [16], which may disrupt the neurobiological and reward processes relevant to substance misuse [4]. Further, substantial pre-clinical evidence supports CBD’s neuroprotective and anti-inflammatory properties, which may help mitigate stress-induced or disease-related neuropathology [17, 18]. These mechanisms are relevant to drug reward, mood regulation, neuroinflammation, arousal, and pain modulation, offering a compelling rationale for CBD’s inclusion in integrative treatment approaches. All that said, the link between the known pharmacological effects of CBD and realized clinical response is predominantly speculative at this point and requires additional research to validate. This is a prime example of the extant science being promising, albeit “immature” with respect to the data needed to facilitate clinical implementation.

Human laboratory and clinical studies have consistently demonstrated that CBD lacks intoxicating effects [19, 20] or abuse liability [21] at therapeutic doses. CBD’s safety profile may be especially relevant for conditions where some first-line pharmacological treatments often carry substantial risks of dependence, misuse, or abuse (e.g., opioids for pain or benzodiazepines for anxiety) [22, 23]. In addition, novel low-risk medications are needed to expand the options for vulnerable populations, such as youth and emerging adults at increased risk for psychiatric and substance use disorders. Recent work by Kirkland et al. (2025) demonstrated the feasibility and tolerability of acute CBD in youth with alcohol use disorder, though efficacy was not observed with a single dose [24]. This study underscores the importance of chronic dosing paradigms, pharmacokinetic monitoring, and developmentally tailored endpoints.

A growth in ongoing trials reflects clinical interest in CBD for psychiatric and substance use disorders [25–29]. There is increasing demand for head-to-head comparisons with first-line psychiatric medications, such as selective serotonin reuptake inhibitors (SSRIs) or atypical antipsychotics. Some researchers advocate for CBD as an adjunctive therapy, potentially enhancing treatment response or mitigating adverse effects. There is also a burgeoning interest in CBD as a potential therapeutic in the treatment of psychosis, with some clinical studies showing potential benefit as an adjunctive treatment for bipolar disorder or schizophrenia, but a lack of evidence in others[30–33].

In pediatric and adolescent psychiatry, where pharmacological options are limited and often poorly tolerated, CBD could offer a novel path forward. For example, in autism spectrum disorder (ASD), early case reports suggest potential in reducing irritability and self-injury, warranting rigorous exploration [34, 35]. Likewise, in post-concussive syndrome and traumatic brain injury, preclinical models support CBD’s neuroprotective properties [36, 37].

While gaps remain in our understanding of optimal dosing, formulation, and individual variability, these limitations reflect the early stage of CBD science, not its lack of promise. The current regulatory environment, marked by the removal of hemp from the list of controlled substances in many jurisdictions, yet characterized by patchwork and often conflicting local policies, creates unnecessary barriers to clinical research and patient access. For example, an increasing number of local jurisdictions have laws relating to the legality or definition of hemp, allowable amounts of Δ9-THC or other phytocannabinoids in retail CBD-based products, or the allowable format of finished products relative to national or state policies. Overregulation that does not account for product aspects like route of administration, format, and manufacturing methods risks stalling progress on a medication with strong translational potential and few viable alternatives in certain clinical populations.

Taken together, CBD represents a relatively low-risk intervention for which developing neurobiological and clinical data suggest may have therapeutic benefit in the treatment or symptom management of a number of different health conditions that are worthy of continued scientific investment. The priority now should be evidence generation and facilitation of rigorous research.

## Perspective two: the CBD boom outpaces the science: limitations, risks, and premature conclusions

Despite the potential for clinical application across the range of health conditions listed above, the evidence base for using CBD for any health condition outside of pediatric epilepsy simply remains insufficient [38, 39]. Although CBD exhibits pharmacological effects on a number of key receptor systems (as described above), the exact neurobiological mechanism by which CBD could treat any of the number of health conditions of interest listed above has not been elucidated. Of additional concern is that the diversity of CBD product types available to consumers, as well as variable regulatory environments in which they are sold, creates challenges in generalizing the potential therapeutic effects of CBD for any health condition to products/formulations other than the exact product tested in a clinical trial. To date, clinical research on other target therapeutic areas has mostly consisted of laboratory studies in healthy adult populations [e.g., 40]. These studies often use acute dosing paradigms with small, underpowered clinical trials of short duration, or larger observational studies of biased samples [41]. Aside from the sparse clinical evidence, certain regulatory and potential health concerns would need to be resolved prior to the adoption of non-pharmaceutical forms of CBD into clinical medicine.

Currently, the umbrella of “CBD” products includes categories of retail goods that are intended to be inhaled, orally ingested, topically applied or anally/vaginally inserted. Moreover, CBD products vary widely with respect to chemical composition. Industry nomenclature categorizes these products using the terms “full spectrum” (products infused with a raw botanical extract expected to contain small concentrations of delta-9-tetrahydrocannabinol (Δ9-THC) along with other “minor” cannabinoids, terpenoids, and flavonoids), “broad spectrum” (products expected to contain “minor” cannabinoids, terpenoids, and flavonoids, but no Δ9-THC or other intoxicating chemicals), and “isolate” (products infused with CBD alone and no more than trace amounts of other cannabinoids, terpenoids, and flavonoids) [42, 43]. Further, multiple product formats are used within each of these categories, which impact the pharmacokinetics of exposure [44]. The heterogeneity of route of administration, chemical composition, and product format leads to significant diversity of both pharmacokinetics and pharmacodynamics, such that generalizable claims about safety or therapeutic efficacy related to “CBD” use cannot validly be made but rather must be constrained to a specific product.

Given the diversity of CBD product types, it is not surprising that there are mixed results with respect to efficacy for most health conditions for which CBD is considered a promising pharmacotherapy. For example, a recent systematic review and meta-analysis of clinical studies evaluating the effects of CBD on social anxiety disorder, generalized anxiety disorder, post-traumatic stress disorder (PTSD), obsessive-compulsive disorder (OCD), and Tourette syndrome showed moderate therapeutic benefit for pure CBD in social or generalized anxiety disorders, but no effect when combined with Δ9-THC and the authors noted the low quality and quantity of evidence [45]. A review of CBD in the prevention and/or treatment of various types of cancer reported antioxidant and tumor suppression and reduction effects of CBD oil in mechanistic preclinical studies; however, concerns were noted in regard to adverse events, poor quality of evidence, and product heterogeneity that preclude integration into clinical practice [46]. These two examples are representative of the current findings related to the effects of CBD for many health conditions of interest. Further, it is pertinent to note that high placebo response rates in clinical trials, lack of appropriate control groups, exclusion of comparative medications, and the frequent use of biased populations in observational studies (e.g., assessment of long-term CBD product users) also significantly limit interpretation of research results [47].

In addition to CBD product heterogeneity being a concern with respect to generalization of clinical findings, studies have demonstrated poor quality control of CBD products that further limits consumer confidence and the adoption of artisanal CBD products by the medical community. Product surveillance testing studies have repeatedly shown poor dose label accuracy, extreme ranges of CBD concentrations in similar product types, and the presence of potentially harmful contaminants in inhaled, oral, and topical CBD products [42, 48–50]. Improved industry regulations for manufacturing and labeling, accompanied by diligent regulatory oversight, are urgently needed, given that the predominant use of these products is focused on health and treatment of disease (despite the lack of federal health agency approvals for most products).

Though acute administration of CBD is not typically associated with intoxication or impairment of functioning and has a large therapeutic window, there are potential safety risks associated with CBD product use that are often understated or unrecognized. As mentioned above, many retail “CBD” products also contain Δ9-THC with ratios of CBD to Δ9-THC varying substantially [46], and the optimal ratio of CBD to Δ9-THC for a given therapeutic purpose is largely unknown for most health conditions. For example, though most healthy adults exhibit no untoward effects of low-dose Δ9-THC exposure [51], case reports have shown that even small doses of Δ9-THC can cause significant adverse events in some clinical populations [52]. Moreover, chronic exposure to high-dose CBD has been shown to negatively impact hepatic functioning in some cases [53]. This risk of liver injury is related to CBD dose, frequency of use, age, and use of concomitant medications, but can be effectively managed by monitoring liver function and following recommended clinical guidelines [54]. In addition to the potential risk of hepatotoxicity, orally administered CBD has been shown to be a potent inhibitor of multiple CYP450 enzymes that can significantly slow the metabolism of many drugs [55] and recently, a study indicated that oral CBD could exacerbate the adverse effects of Δ9-THC in patients with schizophrenia[56]. Thus, co-use of oral CBD with a variety of psychoactive medications and/or drugs of abuse, including Δ9-THC (in contrast to claims that CBD attenuates the acute effects of Δ9-THC), can increase the risk of medication-related adverse events.

## Integration: Where do we go from here? common ground and future directions

Despite the differences, both perspectives presented above share foundational points of agreement that can be leveraged to balance the promise of CBD with the prudence needed to build the evidence base. There is consensus that CBD has therapeutic potential in select psychiatric and neurological disorders as well as other areas of medicine. Its non-intoxicating nature, low abuse liability, and favorable safety profile make it an appealing candidate in populations where conventional pharmacotherapies fall short. However, the recognition that CBD has the potential to significantly interact with other medications, exacerbate the effects of co-administered oral Δ9-THC, and potentially harm liver function necessitates that therapeutic use of CBD include the guidance of a health care provider.

**Fig. 1:**
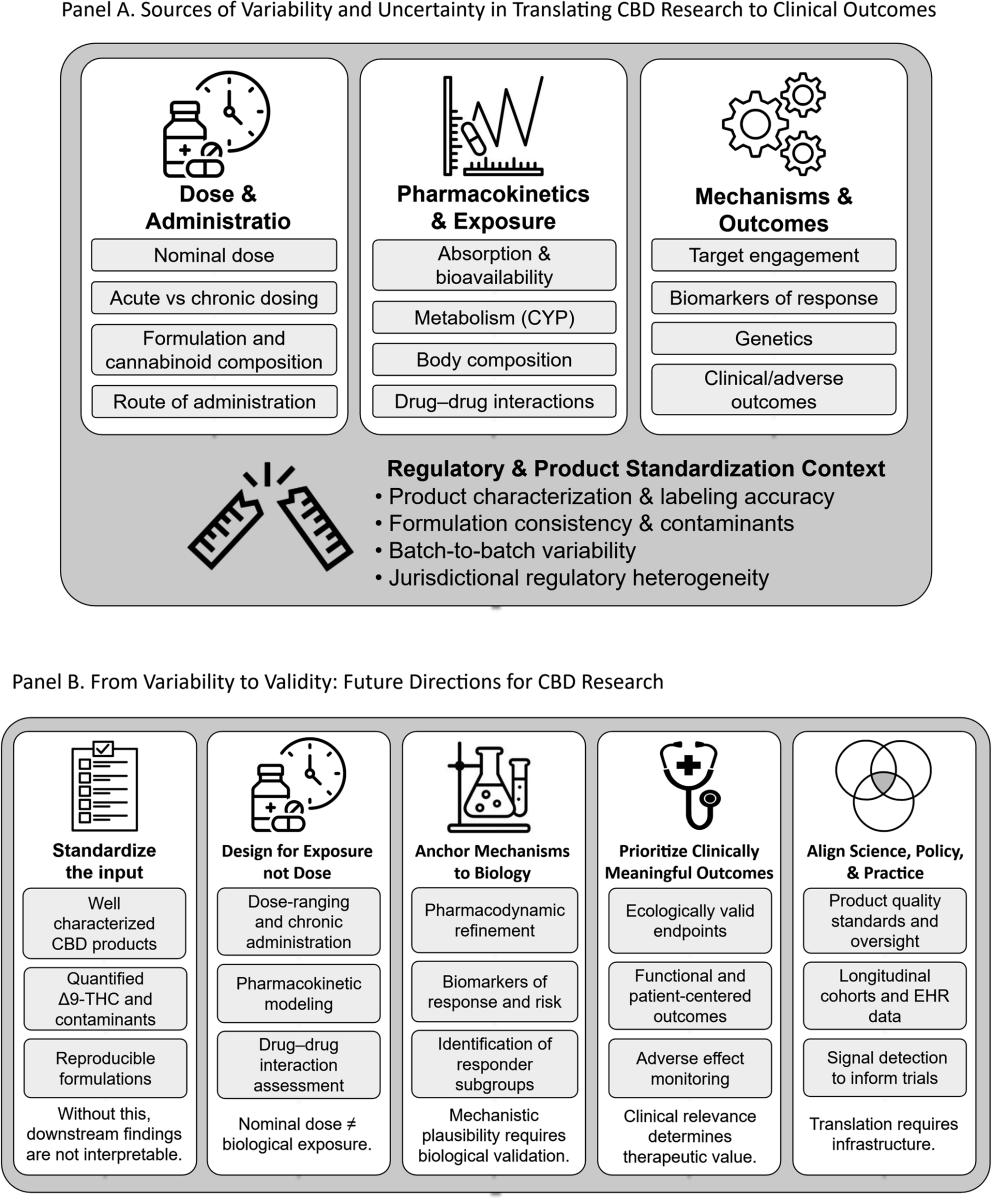
Panel A depicts sources of variability and uncertainty in translating CBD research to clinical outcomes. **A** Sources of Variability and Uncertainty in Translating CBD Research to Clinical Outcomes. Key concepts demonstrating how variability at the levels of dosing, pharmacokinetics, and mechanistic translation contributes to inconsistent findings in the CBD literature. The model highlights the need for integrated study designs that link administered dose to biological exposure and clinically meaningful outcomes. **B** Figure 1, Panel B depicts a roadmap illustrating strategies to reduce variability and improve clinical translation in CBD research. Aligning standardized and well-characterized products, exposure-informed trial design, biomarker-anchored mechanisms, clinically meaningful outcomes, and regulatory oversight is essential for defining CBD’s therapeutic utility.

Across domains, evidence converges on substantial complexity in CBD’s dose–response properties, pharmacokinetics, and mechanisms of action, while key uncertainties limit confident translation to clinical use. Available data suggest that CBD does not exhibit simple linear dose–response relationships, with effective dose ranges likely varying by outcome, population, and dosing duration. At the same time, pronounced interindividual variability in pharmacokinetics, driven by formulation, route of administration, metabolism, and body composition, means that the administered dose often poorly predicts systemic or central nervous system exposure. Although preclinical studies demonstrate biologically plausible mechanisms relevant to neuropsychiatric outcomes, many observed effects occur at exposure levels that are difficult to map onto typical human use, complicating the interpretation of translational relevance. Together, these factors help explain inconsistent findings across studies and underscore the need for research approaches that explicitly integrate dose-response modeling, pharmacokinetic characterization, and mechanistic biomarkers to clarify when, for whom, and under what conditions CBD may confer benefit (See Fig. 1, Panel A).

There is also agreement that the current state of the CBD market in many jurisdictions is dominated by poorly manufactured products, misinformation, and inadequate quality control. This poses significant public health risks. Though these concerns do not extend to pharmaceutical-grade CBD products or CBD products produced under tight regulatory control in some jurisdictions, a common call emerges for standardized product labeling that includes quantitation of CBD, Δ9-THC and other cannabinoids, manufacturing and testing practices that assure quality control, and tighter regulatory oversight and enforcement to be universally adopted. Such improvements in standardized labeling and regulatory oversight would also enable more rigorous and transparent reporting of product characteristics in research studies using commercially available CBD products, thereby strengthening reproducibility, interpretability, and cross-study comparability.

Future research priorities should include well-powered, randomized clinical trials designed to address core scientific questions about CBD’s therapeutic potential. As outlined in Fig. 1, Panel B, these trials should employ chronic dosing schedules, ecologically valid outcomes, placebo controls, and, when appropriate, positive controls to establish comparative safety and efficacy. Complementary pharmacokinetic and dose-ranging studies are also needed to better account for interindividual variability in metabolism, body composition, and absorption. In parallel, biomarker-based approaches may help identify patient subgroups and psychiatric phenotypes most likely to benefit from CBD, supporting more precise targeting of clinical development efforts and minimizing unnecessary exposure.

Distinct from these scientific aims, policy and regulatory priorities include improving standards for product quality assurance, including consistency in formulation, labeling, and contaminant testing. Strengthening these standards would substantially enhance the interpretability of real-world evidence and enable more reliable assessment of the health impacts of commercially available CBD products. In this context, large longitudinal cohorts and analyses of electronic health records hold promise for bridging the research, policy, and regulatory gaps by informing clinical decision making related to product selection and use patterns, as well as for identifying high-priority signals that warrant more rigorous, hypothesis-driven drug development and clinical trials [57].

Finally, cross-disciplinary collaboration will be key. Addiction scientists, psychiatrists, pediatricians, pharmacologists, and regulatory experts must work together to shape a research agenda that is both scientifically rigorous and clinically relevant. By fostering transparency, shared standards, and critical engagement, the field can move beyond polarized narratives and toward data-driven consensus.

CBD is neither a miracle nor a menace. It is a compound with intriguing properties, a modest but growing evidence base, and an urgent need for further inquiry. At this crossroads, the choice is clear: proceed not with hype, but with humility and evidence.
